# Catechin-capped gold nanoparticles: green synthesis, characterization, and catalytic activity toward 4-nitrophenol reduction

**DOI:** 10.1186/1556-276X-9-103

**Published:** 2014-03-03

**Authors:** Yoonho Choi, Myung-Jin Choi, Song-Hyun Cha, Yeong Shik Kim, Seonho Cho, Youmie Park

**Affiliations:** 1 College of Pharmacy, Inje University, 197 Inje-ro, Gimhae, Gyeongnam 621-749, Republic of Korea; 2 National Creative Research Initiatives (NCRI) Center for Isogeometric Optimal Design, Seoul National University, 1 Gwanak-ro, Gwanak-gu, Seoul 151-744, Republic of Korea; 3 College of Pharmacy and Natural Products Research Institute, Seoul National University, 1 Gwanak-ro, Gwanak-gu, Seoul 151-742, Republic of Korea

**Keywords:** Green synthesis, Catechin, Gold nanoparticles, Catalysis, 4-Nitrophenol reduction

## Abstract

An eco-friendly approach is described for the green synthesis of gold nanoparticles using catechin as a reducing and capping agent. The reaction occurred at room temperature within 1 h without the use of any external energy and an excellent yield (99%) was obtained, as determined by inductively coupled plasma mass spectrometry. Various shapes of gold nanoparticles with an estimated diameter of 16.6 nm were green-synthesized. Notably, the capping of freshly synthesized gold nanoparticles by catechin was clearly visualized with the aid of microscopic techniques, including high-resolution transmission electron microscopy, atomic force microscopy, and field emission scanning electron microscopy. Strong peaks in the X-ray diffraction pattern of the as-prepared gold nanoparticles confirmed their crystalline nature. The catalytic activity of the as-prepared gold nanoparticles was observed in the reduction of 4-nitrophenol to 4-aminophenol in the presence of NaBH_4_. The results suggest that the newly prepared gold nanoparticles have potential uses in catalysis.

## Background

Recent advances in nanotechnology have resulted in diverse applications of gold nanoparticles (AuNPs) in various research fields. AuNPs are the most stable NPs and are used in novel applications, including as vehicles for drug/gene delivery, catalysts, optical sensors, and imaging and visualization agents [1-3]. In addition, the catalytic properties of AuNPs have been explored, and the AuNPs have been found to exhibit improved catalytic performance compared with that of their bulk counterpart. The catalytic activity of AuNPs has been commonly evaluated using a well-known reaction: the reduction of 4-nitrophenol (4-NP) to 4-aminophenol (4-AP) in the presence of NaBH_4_. 4-NP is an industrial waste and environmental hazard with a long degradation time. Thus, the removal of this component from water is important for public health. The product, 4-AP, is a useful intermediate in the manufacture of antipyretics and analgesics.

Recently, the green synthesis of AuNPs using biological entities as reducing agents has been rapidly replacing chemical methods in which toxic chemicals are utilized. This approach provides numerous benefits, including the high biocompatibility and good water solubility of the resultant AuNPs. Furthermore, the process is eco-friendly and time and cost effective. Plant extracts and pure compounds from plant sources have been demonstrated to be highly effective reducing agents for the synthesis of AuNPs [4].

Catechins are flavanol compounds that are abundant in tea. The biological activities of tea catechins have been extensively reviewed elsewhere [5-8]. Among tea catechins, catechin and epigallocatechin gallate have been used for the synthesis or modification of NPs [9-12]. Ointment of a combination of AuNPs with the antioxidant epigallocatechin gallate and α-lipoic acid accelerated cutaneous wound healing through anti-inflammatory and antioxidant effects [9]. In particular, the topical application of this combined ointment promoted the proliferation and migration of dermal keratinocytes and fibroblasts, which enhanced the restoration of normal skin structures. The same research group has reported that the topical application of the ointment of AuNPs (3 to 5 nm in size) with epigallocatechin gallate and α-lipoic acid effectively promoted wound healing in diabetic mice [10]. The attractive biological activity of epigallocatechin gallate-modified AuNPs is their anticancer activity, which includes efficacy in the treatment of prostate and bladder cancers [11,12]. As an analytical application, catechin-modified TiO_2_-NPs were used as matrices for the analysis of steroid hormones using surface-assisted laser desorption/ionization mass spectrometry [13]. When catechin was bound to the TiO_2_-NP surface, the absorption wavelength increased at 337 nm when compared with that of the unmodified TiO_2_-NPs, which led to an increase in the N_2_ laser absorption efficiencies [13]. As another analytical application, catechin-synthesized AuNPs were used as a nanosensor for the fluorescent detection of lead in water and urine samples [14].

Herein, catechin was used as a reducing agent for the green synthesis of AuNPs at room temperature for 1 h, and the use of other toxic chemicals as reducing agents was avoided (referred to hereafter as catechin-AuNPs). The catechin-AuNPs were characterized using UV-visible spectrophotometry, high-resolution transmission electron microscopy (HR-TEM), atomic force microscopy (AFM), field emission scanning electron microscopy (FE-SEM), and high-resolution X-ray diffraction (HR-XRD). The reaction yield of the synthesis was measured using inductively coupled plasma mass spectrometry (ICP-MS). Furthermore, the catalytic activity of catechin-AuNPs was evaluated on the basis of the reduction of 4-NP to 4-AP in the presence of NaBH_4_.

## Methods

4-Nitrophenol, hydrochloroauric acid trihydrate (HAuCl_4_ · 3H_2_O), sodium borohydride, and (+)-catechin hydrate were purchased from Sigma-Aldrich (St. Louis, MO, USA). Carbon-coated copper grids (carbon type-B, 300 mesh) were purchased from Ted Pella (Redding, CA, USA). The RTESP AFM probe (MPP-11100-10, premium high-resolution tapping mode silicon probe) was obtained from Bruker Nano (Santa Barbara, CA, USA). Mica (grade V-1, 25 mm × 25 mm length, 0.15 mm thick) was purchased from SPI Supplies Division of Structure Probe (West Chester, PA, USA). All the other reagents were of analytical grade. The UV-visible spectra were recorded using a Shimadzu UV-2600 with a quartz cuvette (Shimadzu Corporation, Kyoto, Japan). The HR-TEM images were acquired with a JEM-3010 (JEOL, Tokyo, Japan) operated at 300 kV. The AFM images were obtained using a Dimension® Icon® (Bruker Nano, Santa Barbara, CA, USA) operated under tapping mode. The sample-loaded mica and copper grids were dried in a 60°C oven overnight before the analyses. The FE-SEM images were collected in a JSM-7100 F SEM using an accelerating voltage of 15 kV (JEOL). ICP-MS analysis was performed in an ELAN 6100 (Perkin-Elmer SCIEX, Waltham, MA, USA). The ICP-MS samples were prepared using centrifugation. The centrifugation of catechin-AuNPs was performed at 12,300 × *g* for 40 min, and the supernatant containing the unreacted Au^3+^ was used for ICP-MS analysis. The total concentration of Au^3+^ of the catechin-AuNPs solution was also measured using ICP-MS. The average value of the three measurements was used to determine the yield. For HR-XRD analyses, the catechin-AuNP solution was centrifuged at 12,300 × *g* for 40 min to remove the supernatant. The pellet was pooled and freeze-dried. The freeze-dried samples were prepared with a FD5505 freeze dryer (Il Shin Bio, Seoul, Korea). A Bruker D8 Discover high-resolution X-ray diffractometer (Bruker, Karlsruhe, Germany) equipped with a CuKα radiation source (*λ* = 0.1541 nm) was used in the range of 20° to 90° (2*θ* scale).

The stock solutions of HAuCl_4_ · 3H_2_O (0.5 mM) and catechin (0.5 mM) were prepared using deionized water. Then, 600 μL of HAuCl_4_ · 3H_2_O (0.5 mM) was placed in a 5-mL glass vial with 200 μL of deionized water, and catechin (0.5 mM, 200 μL) was subsequently added to this solution. The reaction mixture was then further incubated under ambient temperature (26°C) for 1 h. The synthesis of gold nanoparticles was monitored through the acquisition of UV-visible spectra.

To evaluate the catalytic activity of the catechin-AuNPs, the reduction of 4-NP to 4-AP in the presence of NaBH_4_ was performed. The catalytic reduction of 4-NP was conducted in aqueous solution under ambient temperature (26°C), and UV-visible spectra were measured in a quartz cuvette. The 4-NP solution (899.9 μL, 0.15 mM) was mixed with deionized water (450.1 μL). Then, freshly prepared NaBH_4_ (1.65 mL, 5.5 mM) was added. To this reaction mixture, 1 mL of freshly synthesized catechin-AuNPs was added. UV-visible spectra were recorded at a time interval of 5 min in the range of 200 to 700 nm.

## Results and discussion

### Green synthesis and the yield of catechin-AuNPs

**Figure 1:**
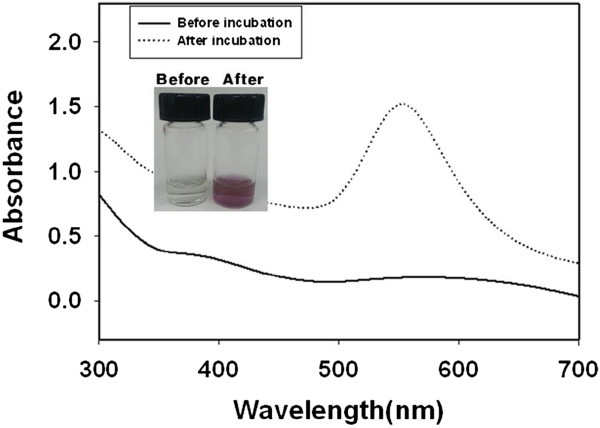
UV-visible spectra of catechin-AuNPs before and after the reaction at room temperature for 1 h.

The color of the solution changed to purple upon reduction of Au^3+^ to Au^0^ by catechin (Figure 1). The characteristic surface plasmon resonance (SPR) band was observed at 553 nm, which indicated the successful synthesis of AuNPs. The reaction proceeded under ambient temperature (26°C) for 1 h, which means the reaction was fast and required minimal energy as well as being eco-friendly. The reaction proceeded very rapidly, as indicated by the color becoming purple (which indicates the reduction of Au^3+^) within 1 min.

In general, the stability of tea catechins is affected by temperature and pH [15,16]. The thermal degradation of catechins is noticeable upon with an increase in temperature. Furthermore, tea catechins are very stable at pH levels less than 4, whereas the stability of catechins decreases in alkaline solutions. In terms of the stability point, the reaction conditions that were used in the present research minimized the thermal and pH degradation of catechin, which may have facilitated the reaction. The pH of the HAuCl_4_ solution was less than 4, and no other reagents were added to adjust the pH. In addition, the reaction was performed under ambient temperature (26°C) without the input of any external energy.

We determined the yield of the reaction by measuring the concentration of unreacted Au^3+^ using ICP-MS. After the sample was subjected to centrifugation, the purple color disappeared in the supernatant, which indicated that the AuNPs were effectively separated from the unreacted Au^3+^. The yield was 99.1% indicating that the reaction occurred very efficient.

### HR-TEM images

**Figure 2:**
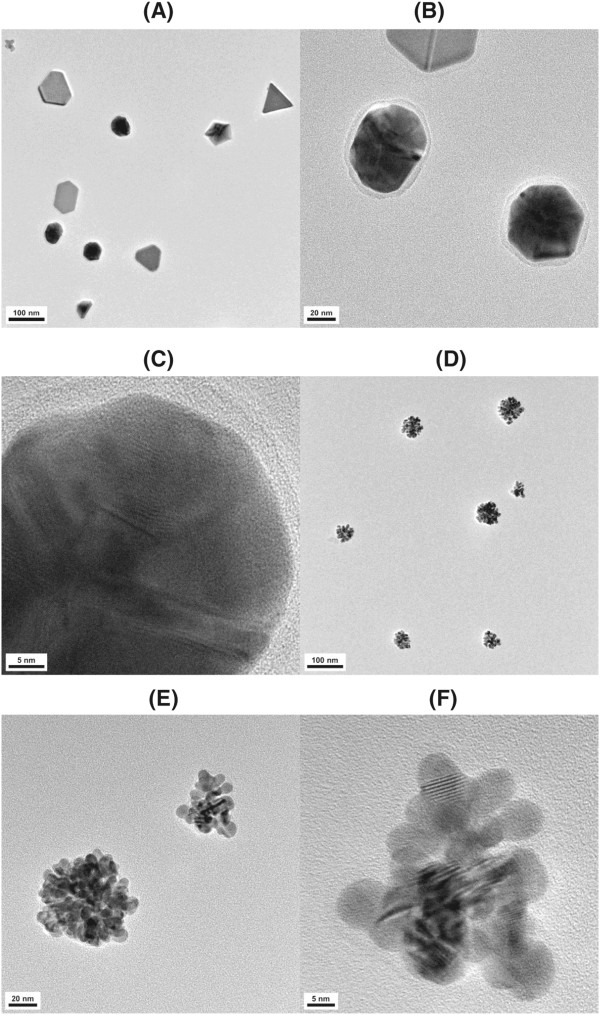
**HR-TEM images of freshly green-synthesized AuNPs.** The scale bar represents **(A)** 100 nm, **(B)** 20 nm, **(C)** 5 nm, **(D)** 100 nm, **(E)** 20 nm, and **(F)** 5 nm.

HR-TEM images generally provide information regarding the size, shape, and dispersion state of NPs. As illustrated in Figure 2, various shapes of AuNPs were synthesized, including spherical, triangular, pentagonal, hexagonal with nonequilateral edges, irregular, and urchin-like shapes. A high-magnification image of several AuNPs is presented in Figure 2B. All the AuNPs were surrounded by shells, which were also observed in the AFM and FE-SEM images. The width of the shells was measured to be 5.41 ± 0.21 nm from ten measurements taken from Figure 2B. A lattice fringe is clearly observed in Figure 2C, which indicates the crystalline nature of the synthesized AuNPs. In addition, the shell is also clearly observed in Figure 2C. Another interesting shape is the urchin-like shape observed in Figures 2D,E,F. The high-magnification image in Figure 2F clearly reveals the lattice fringes in the urchin-like shapes, which also confirms the crystalline nature of the AuNPs. The crystalline structure of the catechin-AuNPs will be further discussed in the HR-XRD section. Lu and co-workers reported the synthesis of biocompatible and urchin-like-shaped AuNPs with excellent surface-enhanced Raman scattering (SERS) activity [17]. Notably, Wang and co-workers observed that Au urchin-like shapes exhibit much greater SERS activity compared to that of Au microspheres [18].

**Figure 3:**
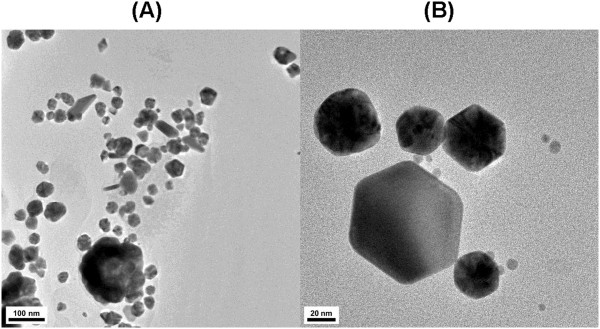
**HR-TEM images of 6-day-old AuNPs.** The scale bar represents **(A)** 100 nm and **(B)** 20 nm.

We hypothesized that the shells that surrounded the AuNPs in Figure 2 might be catechin playing a role as a capping and stabilizing agent. To test this hypothesis, catechin-AuNPs were stored at room temperature for 6 days. As illustrated in Figure 3, the shells all disappeared, and mostly amorphous-shaped AuNPs were observed; these AuNPs exhibited a tendency to aggregate. Thus, we concluded that the shells are catechins playing an essential role in stabilizing the colloidal AuNPs.

### AFM and FE-SEM images

**Figure 4:**
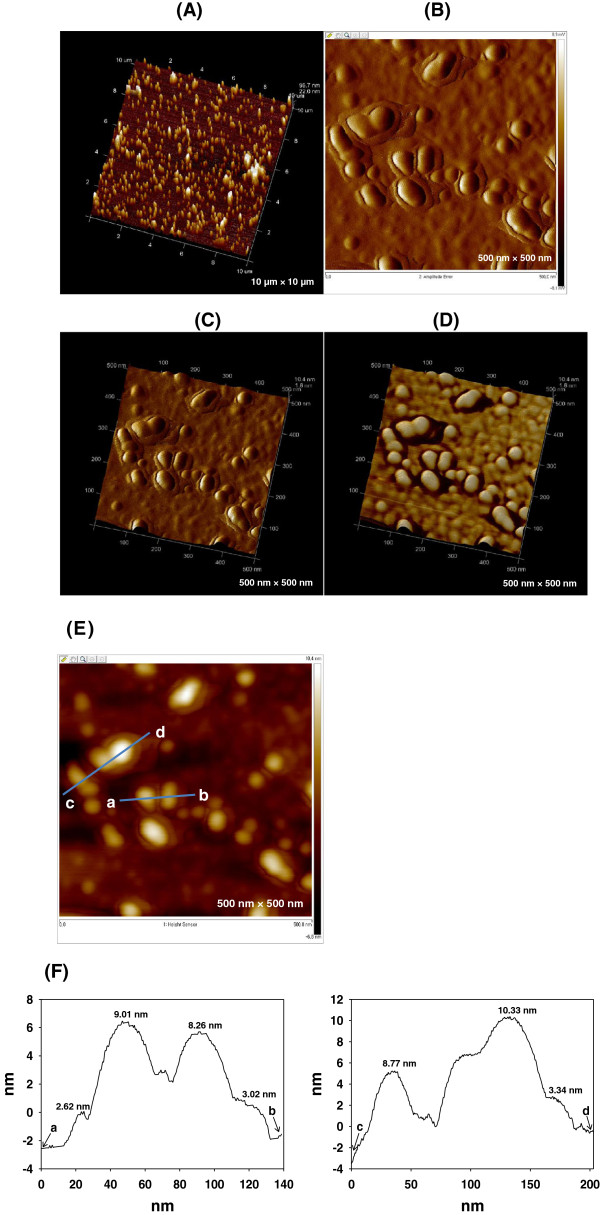
**AFM images. (A)** 3-D height (10 μm × 10 μm), **(B)** 2-D amplitude error (500 nm × 500 nm), **(C)** 3-D amplitude error (500 nm × 500 nm), **(D)** 3-D phase (500 nm × 500 nm), **(E)** 2-D height (500 nm × 500 nm), and **(F)** section analysis of lines a-b and c-d in image **(E)**.

**Figure 5:**
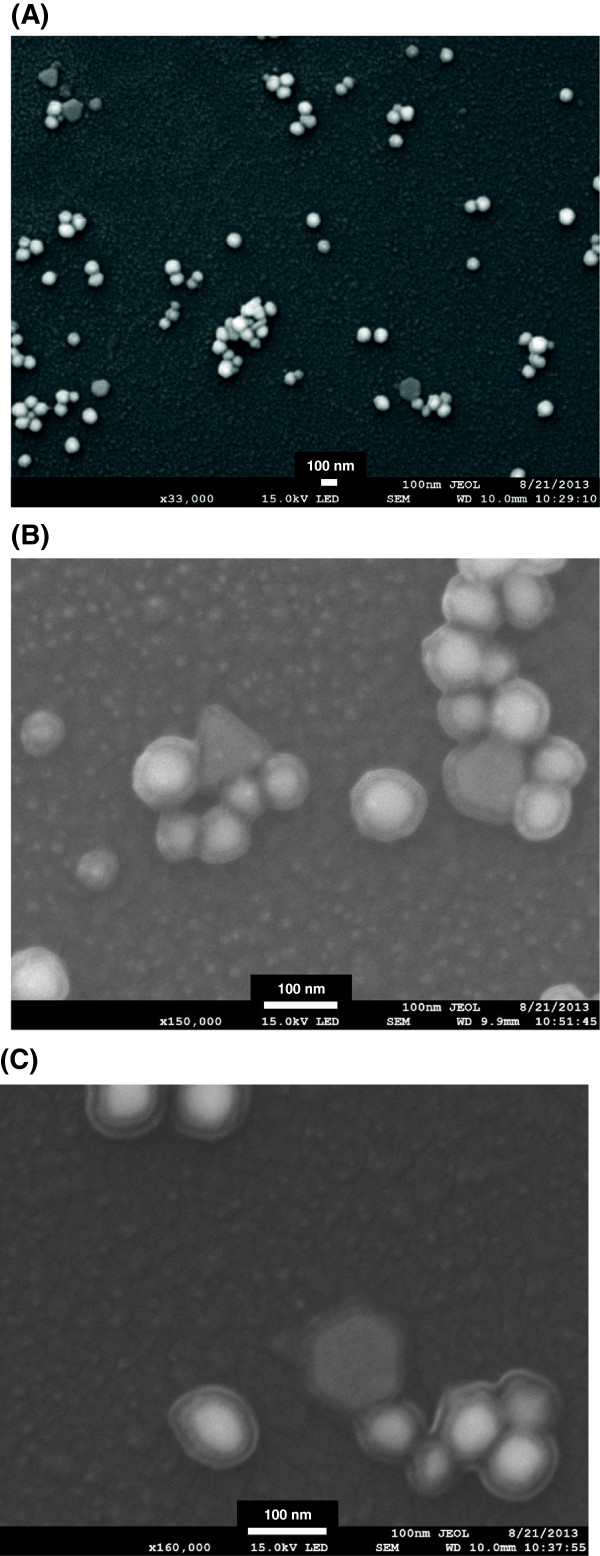
**FE-SEM images.** The magnifications of the images are **(A)** × 33,000, **(B)** × 150,000, and **(C)** × 160,000.

The AFM and FE-SEM images provide further information regarding the 3-D structures and topography of the nanostructures. The 3-D height AFM image in Figure 4A clearly shows that the AuNPs were successfully green-synthesized using catechin as a reducing agent. In the height image, the brighter color NPs possess greater heights. As mentioned previously in the HR-TEM section, the shells were also observed in the AFM images. In the 2-D and 3-D amplitude error images, the shells were clearly discernible from the AuNPs (Figures 4B,C). In the 3-D phase images shown in Figure 4D, the light-yellow-colored AuNPs are surrounded by dark-purple-colored shells. The section analysis of lines a-b and c-d in Figure 4E is depicted in Figure 4F. The heights of randomly selected NPs were measured to be 8.26 to 10.33 nm. In addition, the average value of shell height was determined to be 2.99 nm. The FE-SEM images in which all of the AuNPs possessed shells were consistent with the HR-TEM and AFM image analyses (Figure 5).

### XRD analysis

**Figure 6:**
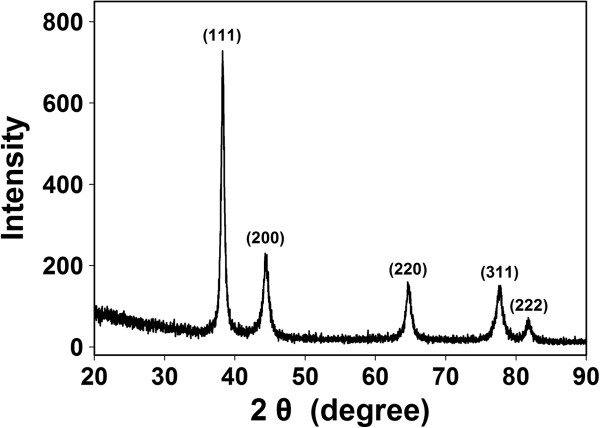
HR-XRD analysis.

The crystalline structure of metallic Au was confirmed by HR-XRD analysis (Figure 6). Intense diffraction peaks were observed at 38.2°, 44.3°, 64.5°, 77.7°, and 81.7°, corresponding to the (111), (200), (220), (311), and (222) planes, respectively, of face-centered cubic (fcc) Au. The predominant orientation was the (111) plane because the most intense peak appeared at 38.2°. The (200)/(111) intensity ratio was 0.32. When compared with the conventional bulk intensity ratio of 0.52, the observed value was low, indicating that the (111) plane was the primary one [19].

The Debye-Scherrer equation (*D* = 0.89*λ*/*W*cos*θ*) was employed to estimate the particle diameter from the (111) peak, and the estimated diameter was approximately 16.6 nm. The definition of each term in the equation is as follows: *λ* is the wavelength of CuKα radiation (0.1541 nm), *W* is the full-width at half-maximum of the (111) peak, *θ* is the diffraction angle, and *D* is the particle diameter.

### Catalytic activity toward 4-nitrophenol reduction

**Figure 7:**
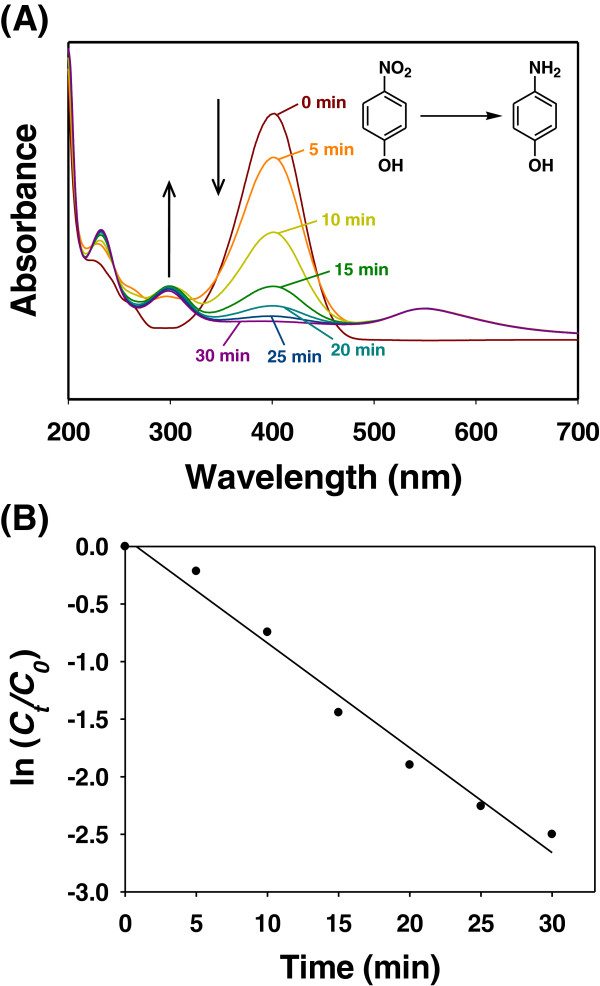
**4-NP reduction by NaBH**_**4 **_**in the presence of catechin-AuNPs catalyst. (A)** UV-visible spectra and **(B)** a plot of ln(*C*_*t*_/*C*_0_) as a function of time (min).

The catalytic activity of green-synthesized AuNPs has been evaluated by other researchers [19-24]. The biological entities used in these studies were cyclodextrins and plant extracts (a glucan of an edible mushroom (*Pleurotus florida*), *Trigonella foenum-graecum*, ayurvedic arishtams, *Anacardium occidentale*, and *Gnidia glauca*). The merit of our method over these reports lies in its energy-saving process, in which no input of external energy is used for the green synthesis of the catechin-AuNPs; in contrast, the other methods used elevated temperatures for the reactions. To evaluate the catalytic activity of the catechin-AuNPs, the reduction reaction of 4-NP to 4-AP in the presence of NaBH_4_ was studied. When NaBH_4_ was added to 4-NP, the color of the solution became yellow, which resulted in a peak at 400 nm in the UV-visible spectrum because of the formation of the 4-nitrophenolate anion. The reaction did not proceed any further in the absence of the catechin-AuNP catalyst. Upon the addition of catechin-AuNPs, the appearance of 4-AP was monitored by the emergence of a peak at 300 nm with a concomitant decrease in the intensity of the peak at 400 nm (Figure 7A). The decreased intensity of the peak at 400 nm and the appearance of the peak at 300 nm were quantitatively monitored by UV-visible spectrophotometry. The approximate time required for the completion of the reaction was 30 min.

The relationship between ln(*C*_*t*_/*C*_0_) and time (min) revealed a linear correlation (*y* = −0.091*x* + 0.071, *r*^2^ = 0.981), where *C*_0_ and *C*_*t*_ are the 4-NP concentration at time 0 and time *t*, respectively (Figure 7B) [21]. The ratio of absorbance, *A*_*t*_/*A*_0_, could be substituted for the ratio of concentration, *C*_*t*_/*C*_0_ (i.e., *C*_*t*_/*C*_0_ = *A*_*t*_/*A*_0_) because the concentration of 4-NP is proportional to its absorbance [21]. On the basis of these results, we determined that the shell did not affect the catalytic activity of the catechin-AuNPs.

## Conclusions

Catechin, which is a potent antioxidant, has been successfully utilized as a green reducing agent for the synthesis of AuNPs. No external energy was necessary during the 1 h reaction, which was simple, fast, energy-saving, and eco-friendly. Together with spherically shaped AuNPs, anisotropic AuNPs with diverse shapes were also observed. The crystalline nature of the AuNPs was confirmed by the resulting HR-XRD peaks and the lattice fringes in the HR-TEM images. Most notably, the capping of AuNPs with catechins was clearly visualized in the microscopic images. The width and height information of the shells was obtained from the HR-TEM and AFM images, respectively. The catechin shells were observed to disappear after the catechin-AuNPs were stored at ambient temperature, during which the aggregation of the AuNPs increased. Thus, catechin plays a role as a reducing agent and is also responsible for the capping of AuNPs. The catalytic activity of catechin-AuNPs for the reduction of 4-NP demonstrated that the newly-prepared AuNPs can be used as a catalyst that is prepared via a green synthesis route.

## Competing interests

The authors declare that they have no competing interests.

## Authors' contributions

YC performed the green synthesis of the catechin-AuNPs. MJC, SHC, and YP characterized the catechin-AuNPs. YSK, SC, and YP supervised the entire process and drafted the manuscript. All authors read and approved the final manuscript.
